# Confidence intervals and sample size planning for optimal cutpoints

**DOI:** 10.1371/journal.pone.0279693

**Published:** 2023-01-03

**Authors:** Christian Thiele, Gerrit Hirschfeld

**Affiliations:** Faculty of Business and Health, University of Applied Sciences Bielefeld, Bielefeld, Germany; Dartmouth College Geisel School of Medicine, UNITED STATES

## Abstract

Various methods are available to determine optimal cutpoints for diagnostic measures. Unfortunately, many authors fail to report the precision at which these optimal cutpoints are being estimated and use sample sizes that are not suitable to achieve an adequate precision. The aim of the present study is to evaluate methods to estimate the variance of cutpoint estimations based on published descriptive statistics (‘post-hoc’) and to discuss sample size planning for estimating cutpoints. We performed a simulation study using widely-used methods to optimize the Youden index (empirical, normal, and transformed normal method) and three methods to determine confidence intervals (the delta method, the parametric bootstrap, and the nonparametric bootstrap). We found that both the delta method and the parametric bootstrap are suitable for post-hoc calculation of confidence intervals, depending on the sample size, the distribution of marker values, and the correctness of model assumptions. On average, the parametric bootstrap in combination with normal-theory-based cutpoint estimation has the best coverage. The delta method performs very well for normally distributed data, except in small samples, and is computationally more efficient. Obviously, not every combination of distributions, cutpoint optimization methods, and optimized metrics can be simulated and a lot of the literature is concerned specifically with cutpoints and confidence intervals for the Youden index. This complicates sample size planning for studies that estimate optimal cutpoints. As a practical tool, we introduce a web-application that allows for running simulations of width and coverage of confidence intervals using the percentile bootstrap with various distributions and cutpoint optimization methods.

## Introduction

Cutpoints are frequently used to classify continuous test results, e.g., when individual blood levels are flagged as elevated or when answers to questionnaires are used to screen for mental disorders. Similarly, guidelines on evaluating diagnostic tools often include a stage where it is tested whether individuals with test results above a certain threshold are more likely to have a particular disease than those with results below the cutpoint [1]. Many studies try to empirically determine optimal cutpoints concerning some criterion, such as the Youden Index. However, these studies tend to give vastly different results. For example, a recent review of studies into the diagnostic utility of the Beck Depression Inventory [2] identified 27 studies that empirically determined 17 different optimal cutpoints between 7 and 27.

One reason for that is the varying sample size of these studies, ranging from 112 participants and only six positive cases to 582 participants and 124 positive cases. The sample size affects the reliability of the results and should be taken into account when comparing the different results. Unfortunately, researchers only very rarely use methods to estimate the variability of the cutpoints they identify as optimal and it is often unclear if a required sample size for achieving reliable estimates was determined when a study was planned.

The problem of estimating the variability of optimal cutpoints is complicated by various ways to estimate the optimal cutpoint itself. The most widely-used method is the simple empirical method of calculating sensitivity (*Se*) and specificity (*Sp*) at all possible cutpoints and determining the cutpoint as optimal that has the highest sum of these two. This is analogous to maximizing the Youden index *J*, where *J* = *Se* + *Sp* − 1, or to minimizing the total misclassification error equal to 1 − *J*. By calculating *Se* and *Sp* at various possible cutpoints and plotting 1 − *Sp* on the x-axis versus *Se* on the y-axis, the well known ROC curve can be generated. Graphically, the optimal Youden-index is achieved by the point on the ROC curve with the maximum vertical distance to the diagonal.

However, past research has shown that this nonparametric empirical method of selecting an optimal cutpoint has the highest variance and bias compared to parametric cutpoint estimation methods that correctly assume normal distributions and cutpoint estimation methods that use smoothing procedures [3–5].

Several methods have been proposed to determine confidence intervals for the Youden-index itself or for optimal cutpoints that optimize the Youden Index [6–9]. While most of these, such as bootstrapping, require the complete raw data to construct confidence intervals, the delta method only requires descriptive statistics, i.e., sample sizes, means, and standard deviations for positive and negative cases [10]. Since these are reported in most studies that determine optimal cutpoints, this method could be used to estimate the variance of optimal cutpoints even for studies that did not do so in the first place.

We additionally propose to test the parametric bootstrap to simulate the distribution of optimal cutpoints, since it also relies on assumed parameters of the underlying distributions of positive and negative cases and might be applicable to a wider range of metrics and cutpoint optimization methods. Finally, to put the performance in perspective, these two methods that are based on descriptive statistics will be compared to a standard percentile bootstrap based on the complete raw data. We evaluate the delta method and the parametric bootstrap for constructing confidence intervals for both an empirical and a parametric cutpoint estimation method.

In summary, the present study aims to discuss the following five questions. First, how can confidence intervals for optimal cutpoints be calculated post-hoc based on descriptive statistics? Second, does the coverage of these confidence intervals depend on the cutpoint estimation method? Third, since some methods for constructing confidence intervals and for estimating cutpoints rely on distributional assumptions, what if these assumptions are violated? Fourth, can the methods for calculating confidence intervals be used to plan sample sizes for studies that estimate optimal cutpoints?

## Methods

The following describes the different methods we used to calculate optimal cutpoints and their confidence intervals. After that, we describe the parameters of the simulation study. All analyses were run using the open-source programming language R 4.1.2 [11] and the packages boot 1.3–28 [12], cutpointr 1.1.1 [13], patchwork 1.1.1 [14], and tidyverse 1.3.1 [15].

### Cutpoint estimation

#### Nonparametric empirical method (EMP)

A nonparametric method to estimate the optimal cutpoint $\hat{c^{*}}$ is to empirically assess the sensitivity and specificity at all possible cutpoints, picking the cutpoint leading to the maximal sum of the metric values (EMP). This is analogous to determining the cutpoint $\hat{c^{*}}$ that maximizes *J* by constructing the empirical cumulative distribution functions (cdf) as estimates of the actual cdf of the diseased and healthy populations, *X* and *Y* respectively [5]:

$${\hat{G}}_{d}\left(c\right)=\frac{1}{m}\sum_{i=1}^{m}I\left(x_{i}\leq c\right),\;{\hat{F}}_{h}\left(c\right)=\frac{1}{n}\sum_{i=1}^{n}I\left(y_{i}\leq c\right)$$

where *I*(*u* ≤ *c*) is an indicator function that is 1 if *u* ≤ *c* and 0 otherwise. *G*_*d*_ and *F*_*h*_ are the distribution functions of the diseased and healthy populations, respectively. The Youden Index is thus

$$\hat{J}=max_{c}\left\{\hat{F_{h}}\left(c\right)-{\hat{G}}_{d}\left(c\right)\right\}\;c\in \left\{x_{1},\ldots ,x_{m},y_{1},\ldots ,y_{n}\right\}.$$

Computing *J*, which is a function of sensitivity and specificity, over all distinct values of *c* with this method results in a function that is analogous to the ROC-curve, which also is a function of sensitivity and specificity. Following Fluss [5], we use the midpoints of the optimal cutpoints and the next highest observation as estimates of c*.

#### Normal method (N)

A parametric method to calculate an optimal cutpoint is to assume that the observations of *X* and *Y* are independently normally distributed as $X∼N(\mu_{D},\sigma_{D}^{2})$ and $Y∼N(\mu_{H},\sigma_{H}^{2})$. The cutpoint that maximizes *J* can be calculated parametrically as

$$c^{*}=\frac{\left(\mu_{D}\sigma_{H}^{2}-\mu_{H}\sigma_{D}^{2}\right)-\sigma_{H}\sigma_{D}\sqrt{{\left(\mu_{H}-\mu_{D}\right)}^{2}+\left(\sigma_{H}^{2}-\sigma_{D}^{2}\right)log\left(\sigma_{H}^{2}/\sigma_{D}^{2}\right)}}{\left(\sigma_{H}^{2}-\sigma_{D}^{2}\right)}$$

and as c* = (*μ*_*D*_ + *μ*_*H*_)/2 if the variances *σ*_*D*_ and *σ*_*H*_ are equal. In practice, the unknown parameter values are substituted by the sample statistics. This approach represents the Normal method (N) [5].

#### Transformed-normal method (TN)

Often, the diseased and healthy samples are not normally distributed but can be transformed to normality using a Box-Cox type of transformation *t*:

$$t\left(y\right)=y^{\left(\lambda \right)}=\{\begin{array}{cc} (y^{\lambda }-1)/\lambda & \lambda \neq 0\\ log(y) & \lambda =0 \end{array}$$

After transforming the distributions of the diseased and healthy samples to (approximate) normality, N as defined above can be employed and the resulting cutpoint can be transformed back to the original scale by applying the reverse of *t*. This represents the Transformed Normal method (TN) [5].

A difficulty lies in estimating a common $\hat{\lambda }$ for the diseased and healthy samples. Here, we use the approach developed by Zou et al. [16]. It consists of constructing a profile log-likelihood function assuming a binormal model that can be maximized numerically to obtain $\hat{\lambda }$. The profile log-likelihood is

$$\begin{array}{cc} l(\lambda |x_{1},\ldots ,x_{m},y_{1},\ldots ,y_{n}) & =-m*log\left(s_{x}^{\prime }\right)-n*log\left(s_{y}^{\prime }\right)\\ & +\left(\lambda -1\right)[\sum_{i=1}^{m}log\left(x_{i}\right)+\sum_{j=1}^{n}log\left(y_{j}\right)]+c \end{array}$$

where *c* is a constant, *x*_*i*_ and *y*_*j*_ are the diseased and healthy samples, $s_{x}^{\prime }$ and $s_{y}^{\prime }$ are the sample standard deviations of the transformed diseased and healthy samples, and *m* and *n* are the sizes of the diseased and healthy samples, respectively.

Additionally, it should be noted that situations can arise in which a back-transformation into the original scale is impossible using the Box-Cox transformation as above and its inverse. For example, if λ = −2 and the transformed value is 4, the inverse Box-Cox transformation (λ*y + 1)^(1/λ)^ is undefined. Generally, a suitable transformation method should be selected for the data at hand, as there are alternatives to the classic Box-Cox transformation [17].

### Methods to determine confidence intervals for the optimal cutpoint

#### Nonparametric bootstrap (BP)

A basic method for estimating a confidence interval that has shown to be applicable in a wide variety of situations is the nonparametric percentile bootstrap (BP) [18]. It should be kept in mind that BP is included mainly as an easy-to-understand and easy-to-implement reference method since the main interest lies in methods that can be computed post-hoc based on descriptive statistics alone. For BP, the complete data would have to be accessible. BP resamples the data *B* times with replacement. The resampling is stratified per class to avoid the case of bootstrap samples that do not contain both classes. In every bootstrap sample ${\hat{c}}_{j}^{*}$ is calculated (*j* = 1, …, *B*) and the 1 − *α* confidence interval for c* then ranges from the *α*/2 to the 1 − *α*/2 percentile [10]. BP was run with *B* = 1, 000 bootstrap repetitions.

#### Parametric bootstrap (PN)

An alternative method for post-hoc estimation of confidence intervals is the parametric bootstrap, which proceeds by iteratively simulating data sets for cutpoint calculation. For this method, distributional assumptions are necessary to generate the data sets. In the following, we will consider the cases of normally and lognormally distributed data. Denote the assumed distributions of marker values by *G* and *F* for the diseased and healthy samples, respectively. Then, simulated observations *x*_1,1_, …, *x*_*mr*_ and *y*_1,1_, …, *y*_*nr*_ with sample sizes per class of *m* and *n* are generated with repetitions *r* = 1, …, *R*, where *x* ∼ *G* and *y* ∼ *F*. Subsequently, the cutpoint calculation is carried out in every simulated sample via N, TN or EMP, yielding *R* estimated cutpoints $\hat{c_{r}^{*}}$. Confidence intervals can be obtained as in BP by calculating the *α*/2 and (1 − *α*/2) percentiles. Thus, the algorithm proceeds as follows:

1. Define the distributions *F* and *G*
2. For *r* = 1, …, *R*
  - Generate simulated samples *x*_1,1_, …, *x*_*mr*_ ∼ *G* and *y*_1,1_, …, *y*_*nr*_ ∼ *F*
  - Estimate cutpoint ${\hat{c}}_{r}^{*}$
3. Compute the *α*/2 and 1 − *α*/2 percentiles of the *R* obtained ${\hat{c}}_{r}^{*}$ which then constitute the 100*(1 − *α*)% confidence interval of c*

The parametric bootstrap was run with *R* = 1, 000 repetitions.

#### Parametric bootstrap log-normal (PLN)

If the parametric bootstrap is used in conjunction with TN, the sample mean $\bar{x}$ and sample standard deviation *s* are used to simulate lognormally distributed data sets with $\mu =log(\frac{{\bar{x}}^{2}}{\sqrt{s^{2}+{\bar{x}}^{2}}})$ and $\sigma =\sqrt{log(\frac{s^{2}}{{\bar{x}}^{2}}+1)}$ and normally distributed data sets with $\mu =\bar{x}$ and *σ* = *s*′ if used with N. Also, the empirical method can be used after simulating both normally and lognormally distributed data. Again, the parametric bootstrap for lognormal data was run with *R* = 1, 000 repetitions.

#### Delta method (DN and DLN)

It has been shown that the delta method is suitable for constructing confidence intervals for *J* and c* [10]. After determining the optimal cutpoint using N, a confidence interval can be constructed around that value using normal theory and

$$Var\left(\hat{c}\right)={\left(\frac{\delta c}{\delta \mu_{x}}\right)}^{2}Var\left(\hat{\mu_{x}}\right)+{\left(\frac{\delta c}{\delta \sigma_{x}}\right)}^{2}Var\left(\hat{\sigma_{x}}\right)+{\left(\frac{\delta c}{\delta \mu_{y}}\right)}^{2}Var\left(\hat{\mu_{y}}\right)+{\left(\frac{\delta c}{\delta \sigma_{y}}\right)}^{2}Var\left(\hat{\sigma_{y}}\right)$$

with

$$\begin{array}{c} (\frac{\delta c}{\delta \mu_{y}})=\frac{-1\pm ab{\left(rad\right)}^{-1/2}}{b^{2}-1}\\ (\frac{\delta c}{\delta \mu_{x}})=\frac{b^{2}\pm ab{\left(rad\right)}^{-1/2}\left(-1\right)}{b^{2}-1}\\ \left(\frac{\delta c}{\delta \sigma_{y}})=\frac{2ab}{{\left(b^{2}-1\right)}^{2}\sigma_{x}}\pm [\frac{\left(-b^{2}-1\right){\left(rad\right)}^{1/2}}{{\left(b^{2}-1\right)}^{2}\sigma_{x}}+\frac{\sigma_{y}b{\left(rad\right)}^{-1/2}}{b^{2}-1}\left(ln\left(b^{2}\right)+1-b^{-2}\right)\right]\\ \left(\frac{\delta c}{\delta \sigma_{x}})=\frac{-2ab^{2}}{{\left(b^{2}-1\right)}^{2}\sigma_{x}}\pm [\frac{b\left(b^{2}+1\right){\left(rad\right)}^{1/2}}{{\left(b^{2}-1\right)}^{2}\sigma_{x}}+\frac{\sigma_{x}b{\left(rad\right)}^{-1/2}}{b^{2}-1}\left(ln\left(b^{2}\right)+b^{2}-1\right)\right] \end{array}$$

where *a* = *μ*_*y*_ − *μ*_*x*_, *b* = *σ*_*y*_/*σ*_*x*_ and $rad=a^{2}+(b^{2}-1)\sigma_{x}^{2}ln(b^{2})$. After $Var(\hat{c})$ is obtained, we can build confidence intervals using the familiar $\hat{c}\pm z_{\alpha /2}*\sqrt{Var(\hat{c})}$ as shown by Schisterman [10]. This is the delta-normal (DN) method.

As in TN, we can apply a Box-Cox transformation to the data before calculating the sample statistics and $Var(\hat{c})$. The delta-transformed-normal (DLN) method is carried out by calculating a common λ as in TN, transforming the data accordingly, calculating the confidence interval, and back-transforming into the original scale using the inverse of the prior Box-Cox transformation.

### Simulation study of confidence intervals for optimal cutpoints maximizing the youden-index

We performed a simulation study in which we systematically manipulated the sample size, effect size, and distribution to analyze the finite sample properties of the confidence interval methods. Specifically, we used sample sizes of *m* + *n* = 30, 100, 500. The effect size, i.e., the true difference in distributions between healthy and diseased participants, was manipulated via the mean of the diseased group to be equal to *J* = 0.2 or *J* = 0.8. The mean of the healthy group and the standard deviations of both the healthy and diseased group were constant (see Table 1).

**Table 1:** Overview of simulation scenarios with J = Youden-Index, d = diseased population, and h = healthy population.

Distribution	*μ*_*h*_	*σ*_*h*_	*σ*_*d*_	*μ*_*d*_ per *J*	
				0.2	0.8
normal	100	10	10	105.05	125.63
lognormal	2.5	0.5	0.5	2.76	3.78

We included normal and lognormal data. In the scenarios with normal data this led to the optimal cutpoints c* = 102.53 and c* = 112.82, and in the case of lognormal data to c* = 13.88 and c* = 23.11. All possible combinations of the aforementioned parameters with values as in Table 1 allow for 12 different scenarios. All scenarios were run 1000 times each.

Within each scenario, we used the following nine combinations of methods for estimating cutpoints maximizing the Youden-index and for estimating confidence intervals. If a lognormal distribution was used, we applied the assumption of log-normality to both the construction method for the confidence interval and the cutpoint estimation method:

- Delta method (DN) with N cutpoint estimation
- Delta method with Box-Cox transformation (DLN) and TN cutpoint estimation
- Nonparametric bootstrap (BP) with EMP cutpoint estimation
- Nonparametric bootstrap (BP) with N cutpoint estimation
- Nonparametric bootstrap (BP) with TN cutpoint estimation
- Parametric bootstrap assuming normal data (PN) with EMP cutpoint estimation
- Parametric bootstrap assuming log-normal data (PLN) with EMP cutpoint estimation
- Parametric bootstrap assuming normal data (PN) with N cutpoint estimation
- Parametric bootstrap assuming log-normal data (PLN) with TN cutpoint estimation

### Sample size planning for cutpoint estimation

In the planning phase of a study, usually the question arises what sample size is necessary, given a suspected effect size, to reach a significant test result. Analogically, in the case of cutpoint estimation, a certain maximum width of the confidence interval around the estimated cutpoint could be desired, especially if cutpoints are planned to be estimated for multiple subgroups. As an illustrative example, we determined the widths of confidence intervals in different scenarios with different sizes of the groups and different effect sizes. We calculated the interval widths for cutpoints that optimize the Youden-index using the delta method on normally distributed data with *μ*_*h*_ = 100, *μ*_*d*_ = (105, 110, 115, 120, 125) and *sd*_*h*_ = *sd*_*d*_ = (3, 5, 10, 15, 20). The sizes of the healthy and the diseased groups varied from 100 to 900 in steps of 100. We only included scenarios with *n*_*h*_ ≥ *n*_*d*_, *n*_*h*_ + *n*_*d*_ ≤ 1000, Cohen’s d <2, and *n*_*h*_/*n*_*d*_ ≤ 4, leading to 1812 different scenarios. This simulation study will demonstrate the dependency of the confidence interval widths on the scenario parameters. Furthermore, we will go into some practical considerations and introduce a web application that can simulate confidence interval widths and coverage probabilities for various different distributions, different cutpoint optimization methods, and different metrics.

## Results

### Confidence intervals for optimal cutpoints with normally distributed data

**Fig 1:**
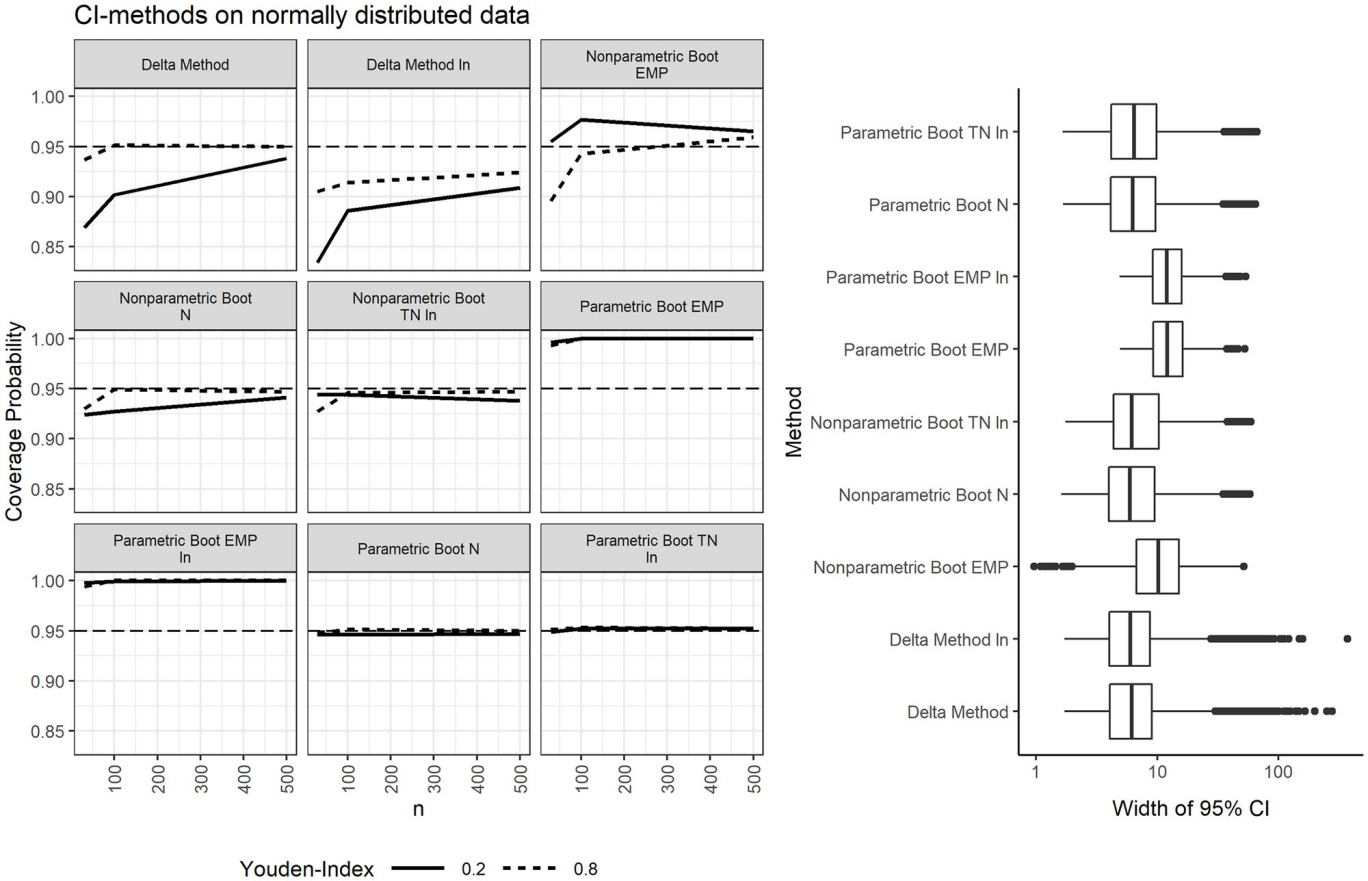
Coverage probabilities and widths of 95% confidence intervals for the optimal cutpoints on normally distributed data.

In scenarios with normally distributed data and N for cutpoint estimation, the parametric bootstrap offers relatively narrow confidence intervals and near-exact coverage of 0.946 to 0.95 over all sample sizes and effect sizes when *α* = 0.05, see Fig 1 and S1 and S2 Tables. The coverage is still near-exact when a Box-Cox transformation (which is not necessary here, as the data is already normal) is applied, with coverages between 0.949 and 0.952.

However, the coverage probabilities are between 0.993 and 1.00 when using EMP in conjunction with the parametric bootstrap. The problem of wide confidence intervals caused by the bootstrap distribution of cutpoints estimated by EMP was already noted by other authors [19]. This distribution is often not normal, not a smooth distribution, and not symmetrical. Instead, these bootstrap distributions often show several local maxima. EMP used in conjunction with the nonparameric percentile bootstrap has coverages varying between 0.896 and 0.977 and seems to converge to slight overcoverage in larger samples. We also tested the normal and basic bootstrap confidence interval from the boot package [12], but with worse results compared to the percentile bootstrap (results not shown).

Confidence intervals based on the delta method are about as narrow as the ones based on the bootstrap methods, but undercovered by several percentage points in most scenarios. Coverage probabilities range from 0.869 to 0.951 and from 0.834 to 0.921 when a Box-Cox transformation is applied. An exception with near-exact coverage of the delta method are scenarios with *m* + *n* ≥ 100 and *J* = 0.8. Generally, the coverage probability of confidence intervals based on the delta method tends to decrease along with the sample size and effect size. The latter observation seems to be in line with prior results [10].

### Confidence intervals for optimal cutpoints with lognormally distributed data

**Fig 2:**
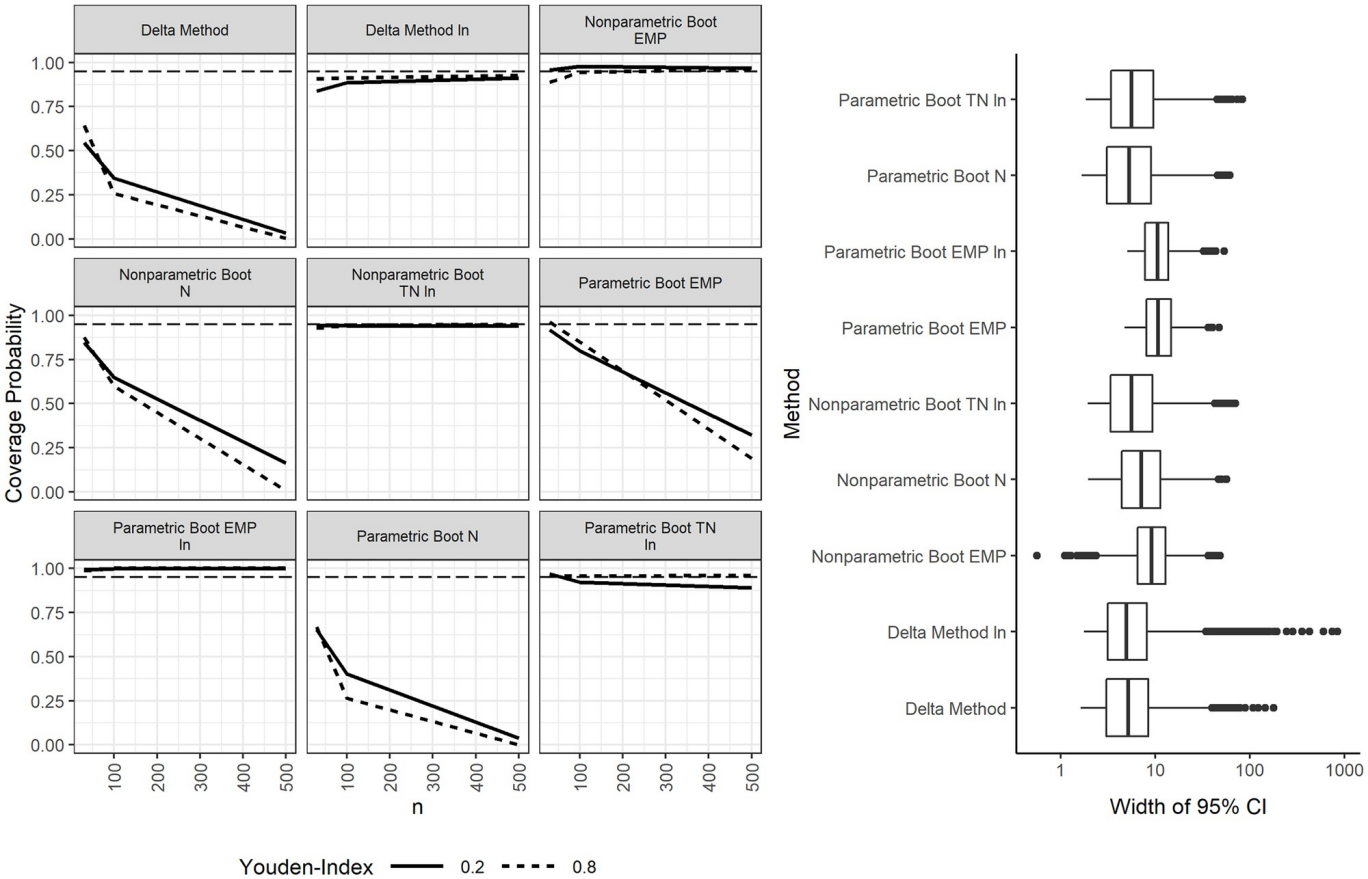
Coverage probabilities and widths of 95% confidence intervals for the optimal cutpoints on lognormally distributed data.

In the case of lognormally distributed data, all methods that assume normally distributed data fail, which was expected (these methods are: the delta method without Box-Cox transformation, the parametric bootstrap assuming normal data for confidence intervals, and N without Box-Cox transformation for cutpoint estimation). Surprisingly, PN with EMP as well as BP with N have coverages near 95% in the smallest samples. This good coverage may however simply be due to relatively wide confidence intervals. All methods assuming normality have coverages below 50% on lognormal data in large samples of *m* + *n* = 500, see Fig 2 and S3 and S4 Tables.

The best method on lognormal data is BP with TN followed by BP with EMP. PLN or DLN with TN tend to produce confidence intervals that are often undercovered by more than five percentage points. This may be due to the fact that the parametric bootstrap and TN rely not only on the estimation of $\bar{x}$ and *s* but also on the estimation of $\hat{\lambda }$. PLN with TN is the best method that can be computed from summary data alone regarding coverage probabilities and produces relatively narrow confidence intervals. The average coverage probability is around the desired level. However, the coverage is too low for small effect sizes and larger samples (≈0.9). The coverage probability of the delta method is also too low at around 0.85 to 0.93, depending on the scenario. Four outliers of interval widths ≥1000 produced by the delta method are not shown in Fig 2. Similar to scenarios with normally distributed data, the coverage probability of the parametric bootstrap with EMP is too high at close to 1.

### Sample size planning for cutpoint estimation

**Fig 3:**
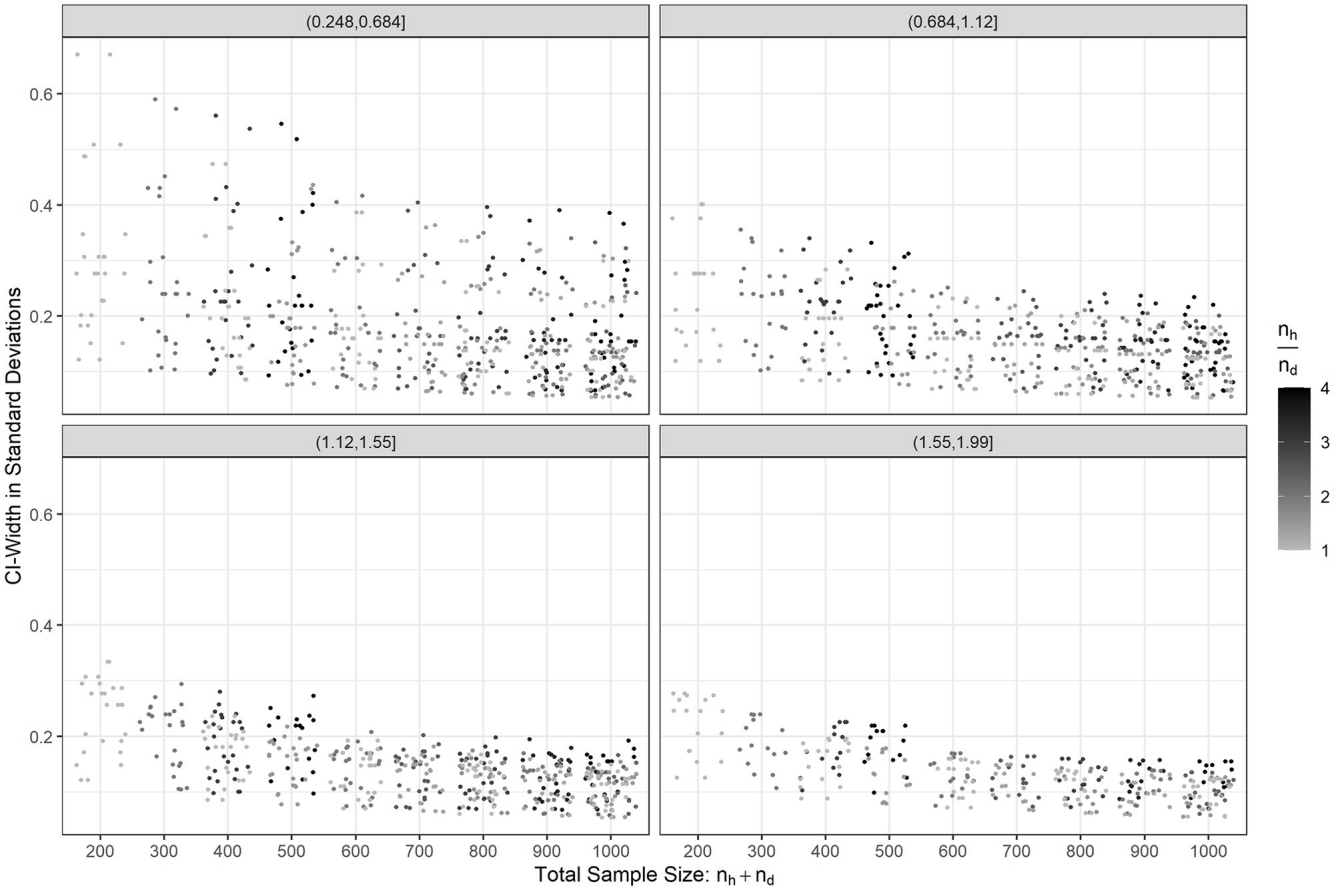
Jitter plot of the width of 95% confidence intervals in terms of the pooled standard deviation in different scenarios with normally distributed data depending on the total sample size, the ratio of the sizes of the two groups and for four ranges of Cohen’s d.

Since the methods for constructing confidence intervals perform very differently depending on the distributions of the marker values, as was seen before, it is not straightforward to carry out a sample size calculation with appropriate precision. Additionally, the formula for the delta method is hard to rearrange in such a way that it gives a simple to understand and simple to use dependence between the parameters of the experiment and the confidence interval width. As an illustration, take Fig 3 which shows results of the confidence interval widths for scenarios with a total sample size between 200 and 1000, Cohen’s d below 2, *n*_*h*_ ≥ *n*_*d*_, and *n*_*h*_/*n*_*d*_ < 4. Generally, the width of the confidence interval decreases with increasing sample size but increases with an increase in *n*_*h*_/*n*_*d*_, all other parameters equal. It also does not necessarily decrease with increasing effect size. Additionally, these results only apply for normally distributed data, where the delta method was shown to perform well.

Given these challenges, we advocate using simulation methods for sample size planning. Based on the cutpoint estimation procedure (the optimization method and the metric), the distributions of the healthy and the diseased sample, and the sample sizes, simulations can be carried out for coverage probabilities and interval widths.

We have made an R Shiny web application based on the cutpointr package available [20] that supports cutpoint estimation and bootstrap validation to estimate the out-of-sample performance, but also two types of simulations: First, to simulate the cutpoint estimation and thus to enable sample size planning given assumed normal, lognormal, or gamma distributions and a cutpoint estimation procedure. Second, to simulate the coverage and width of bootstrapped confidence intervals given assumed distributions, a cutpoint estimation procedure, and the true optimal cutpoint.

The simulation for sample size planning proceeds as follows. A number of simulation runs (preferably at least 1000) is carried out, where in each run a data set based on a specified sample size and distribution, separately for the positive and negative class, is drawn. Then, optimal cutpoints for a specified metric and optimization method are calculated for every sample. Finally, the percentiles of the resulting distribution of optimal cutpoints can be used to estimate the precision at which an optimal cutpoint could be estimated on real data, given a specific optimization procedure and the appropriateness of the chosen distributions. A certain precision of the estimated cutpoint can be arrived at by increasing or decreasing the sample size, which is useful in the planning phase of a study. Details on the simulation procedure and instructions can be found on the web-app.

In the previous sections, we reported coverage probabilities of confidence intervals for normal and lognormal data, two cutpoint optimization procedures (N and EMP), and one metric (the Youden-index) since it is not feasible to report simulation results for all possible combinations of distributions, cutpoint estimation methods, and metrics. The web application enables the user to run a similar coverage simulation for many other combinations of distributions and estimation procedures for confidence intervals based on the percentile bootstrap (BP). Results may be used to ensure that the nonparametric percentile bootstrap exhibits a satisfactory coverage.

The simulation for coverage probabilities of bootstrapped confidence intervals proceeds as follows. As before, a number of simulation runs on simulated data with specified distributions is carried out. From each of these data sets, a number of bootstrap samples (preferably at least 1000) is drawn. Then, an optimal cutpoint is calculated in every bootstrap sample to calculate a confidence interval based on the percentiles of the distribution of these optimal cutpoints. Finally, the confidence intervals from every simulation run can be checked for inclusion of the true optimal cutpoint to estimate the coverage probability of such bootstrap percentile confidence intervals. The true optimal cutpoint must be pre-specified by the user and can be arrived at analytically or via simulation.

The coverage simulation runs a certain number of simulation repetitions, each of which runs a certain number of repetitions of the nonparametric percentile bootstrap for estimating a confidence interval. Both numbers of repetitions can be freely selected. Thus, this simulation consists of an outer simulation loop and an inner bootstrap loop and is computationally expensive. However, many typical simulation setups can be completed within acceptable run times. For example, running 500 repetitions of a coverage simulation for a nonparametric bootstrap with *B* = 1000 bootstrap repetitions and *n* = 100 takes about 15 minutes. The app is open-source so that the code could be adapted to run locally.

The web-application supports various metrics that can be optimized by simple empirical optimization, but also using bootstrapped cutpoints or kernel smoothing. The reader is referred to the web-application itself for more detailed usage instructions and examples.

## Discussion

The first aim of the present study was to evaluate two methods to construct post-hoc confidence intervals for optimal cutpoints that do not require access to the raw data but work on descriptive statistics only. When comparing these methods, we find that, given the assumption of normally distributed data, both methods of interest, the delta method and the parametric bootstrap, are suitable and perform about as well as the nonparametric bootstrap. However, the delta method suffers from low coverage with smaller data sets (*n*_*h*_ + *n*_*d*_ < 100) and low effect sizes, which is in line with previous results [10].

Second, it should be kept in mind that some methods for cutpoint estimation work better with certain methods for estimation of confidence intervals. For example, the delta method as presented in this study only works for estimating the variance of cutpoints that are calculated using N or TN, not using EMP. The percentile bootstrap BP delivered generally robust results, but given the multitude of possible estimation procedures, we propose running simulations for coverage probabilities, if the coverage probability of a certain estimation method for confidence intervals in combination with a certain estimation procedure for cutpoints is unknown. Such simulations can be carried out using the described web-app.

Third, given lognormal data, we were able to estimate confidence intervals with satisfactory coverage only with BP and methods that included a Box-Cox transformation and correctly assumed the lognormal distribution. On the other hand, the confidence interval methods for lognormal data tended to work well on normal data, except for the delta method. Thus, when calculating confidence intervals post-hoc, the coverage of these intervals crucially depends on the correctness of distributional assumptions. It is advisable to calculate post-hoc confidence intervals for multiple plausible scenarios of distributions, if the distributions are unknown, in terms of a sensitivity analysis.

The simulation setup with *J* between 0.2 and 0.8 was chosen for comparability with previous research [5]. It should be noted, however, that many methods for constructing confidence intervals perform worse in scenarios with extremely low or high effect sizes. Indeed, additional simulations have shown that the coverage probabilities with normally distributed data deviate more from the desired coverage with extreme effect sizes. Furthermore, the delta method produced narrow and undercovered confidence intervals for very low effect sizes of around *J* = 0.02 (results not shown).

The fourth aim was to discuss how to determine the necessary sample size for a desired width of the confidence interval around the true cutpoint, which would be useful in the planning phase of a study. Plugging the assumed parameters of the study population into the formula of the delta method allows for estimating the variance and thus for estimating the width of the confidence interval, but depends on the assumption of normality and the N method for cutpoint estimation. As an alternative, we recommend simulating the variance of the cutpoint. We have demonstrated that the nonparametric bootstrap often has good coverage and have made a web application available for simulating the width and coverage of confidence intervals for cutpoints [20].

All in all, our results show that it is feasible to construct confidence intervals for optimal cutpoints using descriptive statistics instead of accessing the raw data, depending on some distributional assumptions. Using either the delta method or the parametric bootstrap to calculate confidence intervals helps to identify studies that give only very unreliable estimates for optimal cutpoints. Furthermore, it opens the possibility of using the delta method or the parametric and nonparametric bootstrap as a starting point for a-priori sample size calculations, since the necessary parameters, such as means and standard deviations for healthy and diseased participants, can sometimes be estimated based on past research.

## Supporting information

S1 Table Coverage probabilities of 95% confidence intervals on normally distributed data when Youden-Index is J = 0.2. (PDF)

S2 Table Coverage probabilities of 95% confidence intervals on normally distributed data when Youden-Index is J = 0.8. (PDF)

S3 Table Coverage probabilities of 95% confidence intervals on lognormally distributed data when Youden-Index is J = 0.2. (PDF)

S4 Table Coverage probabilities of 95% confidence intervals on lognormally distributed data when Youden-Index is J = 0.8. (PDF)
