# Supplementary material for: Confidence intervals and sample size planning for optimal cutpoints
Source: PLoS One. 2023 Jan 3;18(1):e0279693. doi: 10.1371/journal.pone.0279693 (PMC9810177; doi:10.1371/journal.pone.0279693)
Supplement: S1 Table — (PDF) [file pone.0279693.s001.pdf]

Table S1: Coverage probabilities of 95% confidence intervals on normally distributed data when Youden-Index is J = 0.2.

Method	n = 30	n = 100	n = 500
Delta Method	0.869	0.902	0.938
Delta Method ln	0.834	0.886	0.909
Nonparametric Boot EMP	0.955	0.977	0.965
Nonparametric Boot N	0.924	0.927	0.941
Nonparametric Boot TN ln	0.944	0.944	0.938
Parametric Boot EMP	0.996	1.000	1.000
Parametric Boot EMP ln	0.998	0.999	1.000
Parametric Boot N	0.946	0.946	0.947
Parametric Boot TN ln	0.949	0.952	0.952
