# Supplementary material for: Confidence intervals and sample size planning for optimal cutpoints
Source: PLoS One. 2023 Jan 3;18(1):e0279693. doi: 10.1371/journal.pone.0279693 (PMC9810177; doi:10.1371/journal.pone.0279693)
Supplement: S2 Table — (PDF) [file pone.0279693.s002.pdf]

Table S2: Coverage probabilities of 95% confidence intervals on normally distributed data when Youden-Index is J = 0.8.

Method	n = 30	n = 100	n = 500
Delta Method	0.937	0.951	0.950
Delta Method ln	0.905	0.914	0.924
Nonparametric Boot EMP	0.896	0.943	0.959
Nonparametric Boot N	0.930	0.949	0.947
Nonparametric Boot TN ln	0.927	0.946	0.947
Parametric Boot EMP	0.993	1.000	1.000
Parametric Boot EMP ln	0.994	1.000	1.000
Parametric Boot N	0.948	0.951	0.950
Parametric Boot TN ln	0.951	0.953	0.952
