# Supplementary material for: Confidence intervals and sample size planning for optimal cutpoints
Source: PLoS One. 2023 Jan 3;18(1):e0279693. doi: 10.1371/journal.pone.0279693 (PMC9810177; doi:10.1371/journal.pone.0279693)
Supplement: S3 Table — (PDF) [file pone.0279693.s003.pdf]

Table S3: Coverage probabilities of 95% confidence intervals on lognormally distributed data when Youden-Index is J = 0.2.

Method	n = 30	n = 100	n = 500
Delta Method	0.545	0.345	0.032
Delta Method ln	0.838	0.885	0.911
Nonparametric Boot EMP	0.957	0.977	0.966
Nonparametric Boot N	0.846	0.649	0.163
Nonparametric Boot TN ln	0.945	0.942	0.940
Parametric Boot EMP	0.918	0.799	0.323
Parametric Boot EMP ln	0.992	0.999	0.999
Parametric Boot N	0.653	0.402	0.037
Parametric Boot TN ln	0.969	0.921	0.889
