# Supplementary material for: Confidence intervals and sample size planning for optimal cutpoints
Source: PLoS One. 2023 Jan 3;18(1):e0279693. doi: 10.1371/journal.pone.0279693 (PMC9810177; doi:10.1371/journal.pone.0279693)
Supplement: S4 Table — (PDF) [file pone.0279693.s004.pdf]

Table S4: Coverage probabilities of 95% confidence intervals on lognormally distributed data when Youden-Index is J = 0.8.

Method	n = 30	n = 100	n = 500
Delta Method	0.643	0.257	0.003
Delta Method ln	0.907	0.913	0.925
Nonparametric Boot EMP	0.887	0.943	0.962
Nonparametric Boot N	0.876	0.598	0.008
Nonparametric Boot TN ln	0.929	0.945	0.946
Parametric Boot EMP	0.964	0.849	0.190
Parametric Boot EMP ln	0.988	1.000	1.000
Parametric Boot N	0.669	0.264	0.002
Parametric Boot TN ln	0.956	0.957	0.958
